# PGK1 can affect the prognosis and development of bladder cancer

**DOI:** 10.1002/cam4.70242

**Published:** 2024-09-24

**Authors:** Mingde Gao, Haixia Zhu, Haifei Xu, Xiaoxia Jin, Guihua Zheng, Jinfeng Zhu, Chunyan Gu, Xiaolin Wang

**Affiliations:** ^1^ Department of Urology Affiliated Tumor Hospital of Nantong University and Nantong Tumor Hospital Nantong People's Republic of China; ^2^ Department of Central Laboratory Affiliated Tumor Hospital of Nantong University and Nantong Tumor Hospital Nantong People's Republic of China; ^3^ Department of Pathology Affiliated Tumor Hospital of Nantong University and Nantong Tumor Hospital Nantong People's Republic of China; ^4^ Department of Pathology, Nantong Third People's Hospital Affiliated Nantong Hospital 3 of Nantong University Nantong People's Republic of China

**Keywords:** bladder cancer, chemosensitivity, metastasis, PGK1, prognosis, proliferation

## Abstract

**Background:**

Previous studies have demonstrated that the glycolytic enzyme phosphoglycerate kinase 1 (PGK1) can promote tumor development. This study sought to investigate the specific role of PGK1 in bladder cancer (BLCA).

**Methods:**

Public databases and immunohistochemistry assays were utilized to analyze the expression of PGK1 in BLCA and its prognostic significance. Cell proliferation was assessed through CCK‐8 and colony formation assays, while the level of metastasis was evaluated using transwell migration experiments. Additionally, IC_50_ experiments were conducted to assess the impact of PGK1 on cisplatin sensitivity.

**Results:**

The mRNA and protein expression levels of PGK1 were significantly upregulated in BLCA. Cox proportional hazards model analysis revealed that PGK1 and T stage were independent prognostic factors for BLCA patients. Both CCK‐8 and colony assays demonstrated that PGK1 promotes proliferation. Furthermore, a positive correlation was observed between PGK1 and Ki67, a proliferation index. Transwell migration assays confirmed the ability of PGK1 to enhance metastasis. Finally, PGK1 increased the IC_50_ associated with cisplatin treatment in BLCA.

**Conclusion:**

Collectively, these findings suggest that PGK1 may hold clinical value in predicting BLCA prognosis and improving the outcomes of this patient population.

## 1 INTRODUCTION

Bladder cancer (BLCA), also known as bladder urothelial carcinoma, is the most prevalent malignant tumor of the urinary system, with approximately 610,000 new cases and 220,000 deaths reported worldwide each year.^1^ BLCA can be categorized into non‐muscle invasive bladder cancer (NMIBC) and muscle‐invasive bladder cancer (MIBC). It has been estimated that 20%–50% of NMIBC patients progress to MIBC. Additionally, around 25% of newly diagnosed BLCA cases are identified as MIBC,^2^ which exhibits high malignancy and a propensity for metastasis. The 5‐year survival rate for advanced BLCA patients is less than 15%.^3^, ^4^ Cisplatin chemotherapy is the primary approach for treating advanced BLCA patients. However, the overall response rate in clinical settings remains below 50%. Current studies suggest cisplatin resistance contributes to poor treatment efficacy.^5^

Numerous factors have been implicated in cisplatin resistance in tumors. Previous studies have shown that CircRNAs, LncRNAs, and miRNAs regulate cisplatin resistance in BLCA. In addition, DNA, RNA, and protein modifications can modulate cisplatin resistance.^6^, ^7^ Epithelial–mesenchymal transition (EMT) can promote cisplatin resistance.^8^ Not only such, hypoxia and autophagy can also promote the generation of BLCA cisplatin resistance.^9^ Abnormal glycolytic metabolism is significantly associated with cisplatin resistance.^10^, ^11^ Tumor cells predominantly rely on glycolysis for energy production. Therefore, identifying novel targets associated with cisplatin resistance mediated by glycolysis has become a research hotspot to improve the prognosis of BLCA patients. The glycolysis pathway consists of 10 steps, each catalyzed by specific enzymes or enzyme groups. Two enzymes within this pathway are responsible for ATP production, with the initial key enzyme being phosphoglycerate kinase 1 (PGK1).

PGK1 has been linked to cisplatin chemoresistance in ovarian and endometrial cancer.^12^, ^13^ Furthermore, PGK1 has been implicated in regulating EMT to enhance drug resistance in lung cancer.^14^ Overexpression of PGK1 has been shown to promote the proliferation and metastasis of liver cancer cells and is associated with poor prognosis, suggesting its potential carcinogenic role in liver cancer progression.^15^ PGK1 has been reported to be involved in gemcitabine treatment in BLCA.^16^ However, the specific role of PGK1 in BLCA remains unclear. Thus, this study aimed to elucidate the specific functions of PGK1 in BLCA.

## 2 MATERIALS AND METHODS

### 2.1 Bioinformatics analysis

The Cancer Genome Atlas (TCGA) database (https://portal.gdc.com) was used to study the association between PGK1 and EMT‐related proteins in BLCA. Spearman correlation analysis was conducted to analyze the relationship between PGK1 and tumor proliferation index. The Human Protein Atlas (HPA) database (https://www.proteinatlas.org/) was used to analyze whether the protein expression level of PGK1. CCLE datasets (https://portals.broadinstitute.org/ccle/about) were obtained to assess the mRNA expression matrix of PGK1 in different cell lines in BLCA.

### 2.2 Patients and samples

The tissue microarray (TMA) specimens were selected from patients who underwent radical cystectomy or partial cystectomy from June 2012 to March 2018. These patients did not receive neoadjuvant chemotherapy or radiotherapy before the operation. All specimens were from the Affiliated Tumor Hospital of Nantong University & Nantong Tumor Hospital, and the deadline for follow‐up was August 2019.

### 2.3 Immunohistochemistry scoring

The staining of PGK1 (Santa) in TMA was independently evaluated in a double‐blind manner by two pathologists. The following formula was used: PGK1 staining score = the score of staining intensity × the score of stained area percentage. The staining intensity score was 0–3: 0 (no staining), 1 (weak staining = light yellow), 2 (moderate staining = yellow brown), and 3 (strong staining = brown). According to the previous studies, the percentage of dyeing area was divided into four points (1.0%–10%; 2.11%–50%; 3.51%–80%; 4.81%–100%). X‐tile software (released by Yale University) was used to determine the optimal cutoff value of the expression score of PGK1. Based on the staining intensity of Ki67, a staining area percentage of ≤15% was considered indicative of low Ki67 expression, while a percentage of >15% indicated high Ki67 expression.^17^

### 2.4 Cell culture and lentivirus transfection experiments

The cell lines were cultured in RPMI‐1640 medium with 10% fetal bovine serum and 1% penicillin/streptomycin solution in a thermostatic cell incubator. The cells were transfected by lentivirus (Shanghai Heyuan biology): knockdown for PGK1 lentivirus (sh‐TargetSeq: CTGACAAGTTTGATGAGAATG; control‐TargetSeq: CCTAAGGTTAAGTCGCCCTCG) and overexpression for PGK1 lentivirus (over‐PGK1: pSLenti‐SFH‐EGFP‐P2A‐Puro‐CMV‐PGK1‐3xFLAG‐WPRE; control‐empty vector name: GL120 pSLenti‐SFH‐EGFP‐P2A‐Puro‐CMV‐MCS‐3xFLAG‐WPRE).

### 2.5 Western blot

Western blot (WB) followed the routine steps of electrophoresis, membrane transfer, blocking, antibody incubation, and visualization. PGK1 antibody (Santa, 1:500) and β‐Actin antibody (CST, 1:1000) were used for the western blot. The second antibody, mouse (absin, 1:5000) and rabbit (absin, 1:2000), were also utilized for the lab.

### 2.6 Colony formation assay and cell viability evaluation

The lentivirus cells were inoculated on six‐well plates with 500, 1000, and 2000 per hole, respectively, for 10–14 days. 2000 cells/well were added into 96‐well plates to analyze cell proliferation by CCK8. In addition, cisplatin with a gradient concentration of 10–80 μM was added to the plates to assess the IC_50_ by CCK8.

### 2.7 Transwell migration assay

Lentivirus‐transfected cells were seeded in chambers on 24‐well plates at a density of 50,000 cells per well. The upper chamber was filled with 200 μL of 1640 medium, while the lower chamber contained 500 μL of 10% fetal bovine serum. The plates were incubated in a cell incubator for 24 h. Subsequently, the chambers were fixed with 4% paraformaldehyde and stained with crystal violet. After drying, the cells on the outer surface of the chamber were counted under a microscope.

### 2.8 Statistical analysis

The relationship between PGK1 and clinicopathological parameters, as well as the association between PGK1 and Ki67, was assessed using the *χ* ^2^ test in SPSS software (IBM Corp. Released 2017. IBM SPSS Statistics for Windows, Version 25.0. IBM Corp, Armonk, NY). Prognostic significance was evaluated through Cox regression analysis. GraphPad (GraphPad Software version 8, Boston, MA, USA) was employed for data visualization. Statistical analysis involved non‐paired *t*‐tests or paired *t*‐tests, log‐rank tests for generating Kaplan–Meier (KM) survival curves in different groups, and dose–response curves to map the IC_50_ in BLCA cells. All R‐based analyses were performed using R Statistical Software (v4.0.3; R Core Team 2020). A *p*‐value <0.05 was considered statistically significant.

## 3 RESULTS

### 3.1 The expression of PGK1 was significantly upregulated in BLCA tissues

**FIGURE 1:**
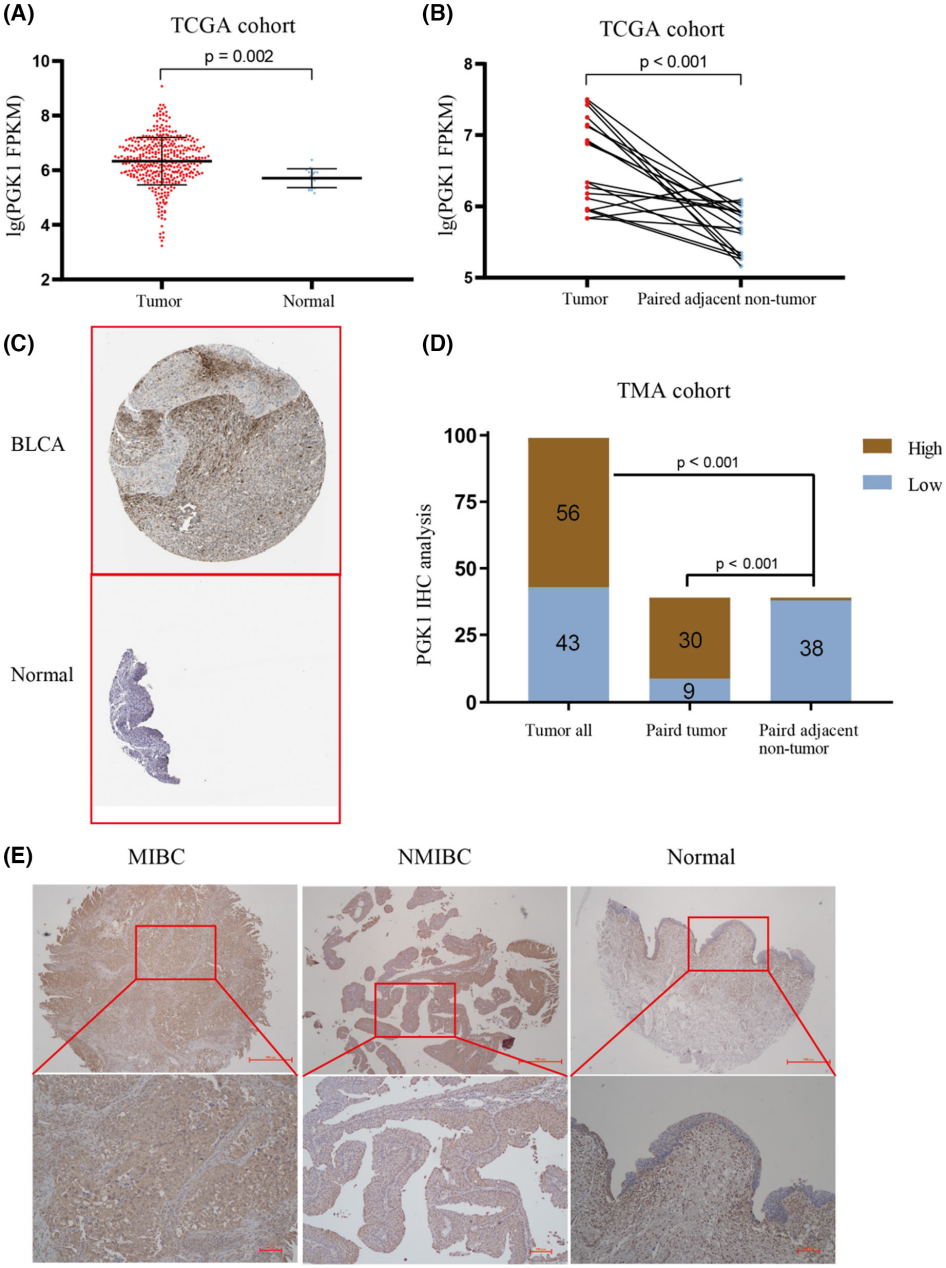
The expression of PGK1 in bladder cancer (BLCA) and adjacent carcinoma. (A) Comparing the expression of PGK1 mRNA in 414 cases of BLCA and 19 cases of para‐cancerous tissues in TCGA database. (B) The expression of PGK1 in 19 matched cancer and adjacent tissues in TCGA database was compared. (C) The protein expression of PGK1 in matched cancer and adjacent tissues in the HPA database was compared. (D) The protein level of PGK1 in 99 cases of BLCA and 39 cases of adjacent tissues were compared by immunohistochemistry. (E) Selecting the representative results of MIBC, NMIBC, and normal tissue.

During analysis of TCGA data, we observed significant upregulation of PGK1 mRNA expression in BLCA tissues (Figure 1A,B). Furthermore, analysis of the HPA database revealed upregulation of PGK1 protein expression in BLCA compared to para‐cancerous tissues (Figure 1C, patient information: male, 66 years old, BLCA, histopathologic grade, ID 1824). Additionally, IHC analysis of the TMA, which consisted of 99 specimens, demonstrated a higher expression of PGK1 in BLCA patients (Figure 1E). The TMA results were sorted and quantified (Figure 1D). Collectively, these findings indicated a high expression of PGK1 in BLCA.

### 3.2 The relationship between PGK1 and clinicopathological features of BLCA

The expression level of PGK1 was evaluated whether it correlated with the clinicopathological characteristics of BLCA patients. We observed a close relationship between different levels of PGK1 expression and tumor grade, vascular invasion, and lymph node metastasis in BLCA patients. However, no relationship was found between PGK1 expression and clinicopathological features such as tumor size, pathological stage, and recurrence (Table 1). These results suggest that PGK1 may be associated with the metastasis of BLCA.

**TABLE 1:** Clinicopathological features of BLCA in relation to the PGK1 expression pattern.

Clinicopathological features		PGK1		*p* value	*χ* ^2^ value
	Total	High	Low		
Gender					
Female	17	10	7	0.837	0.043
Male	82	46	36	–	–
Age					
≤65	39	20	19	0.393	0.731
>65	60	36	24	–	–
Tumor size					
≤5 cm	74	41	33	0.689	0.161
>5 cm	25	15	10	–	–
Tumor stage					
≤ T1	46	23	23	0.22	1.508
≥ T2	53	33	20	–	–
Tumor grade					
Low	34	13	21	0.008*	7.082
High	65	43	22	–	–
Vascular invasion					
Yes	13	11	2	0.029*	4.792
No	86	45	41	–	–
Lymph node metastasis					
Yes	32	24	8	0.011*	6.54
No	67	32	35	–	–
Recurrence					
Yes	22	11	11	0.481	0.496
No	77	45	32	–	–
Distant metastasis					
Yes	10	6	4	0.817	0.053
No	89	50	39	–	–

*Note*: Statistical analyses were carried out using Pearson *χ* ^2^ test.

* *p* value <0.05 was considered statistically significant.

### 3.3 Upregulation of PGK1 was associated with poor prognosis in BLCA

Univariate Cox analysis revealed significant correlations between size, tumor stage, vascular invasion, distant metastasis, PGK1 expression, and the prognosis of BLCA patients. The results of multivariate Cox analysis demonstrated that PGK1 expression and tumor stage were independent prognostic factors (Table 2).

**TABLE 2:** Univariate and multivariate Cox regression analysis for overall survival of patients with bladder cancer (BLCA).

Variates	Univariate analysis		Multivariate analysis	
	*p* value	HR (95% CI)	*p* value	HR (95% CI)
Gender (male vs. female)	0.796	1.175 (0.346–3.993)		
Age (>65 vs. ≤65)	0.369	1.481 (0.628–3.488)		
Tumor size (>5 cm vs. ≤5 cm)	0.012*	3.007 (1.276–7.086)	0.11	2.258 (0.832–6.124)
Tumor stage (≥T2 vs. ≤T1)	0.003*	9.272 (2.158–39.832)	0.049*	4.841 (1.007–23.286)
Tumor grade (High vs. low)	0.214	1.891 (0.692–5.173)		
Vascular invasion (Yes vs. no)	0.005*	3.914 (1.494–10.253)	0.286	1.863 (0.594–5.847)
Lymph node metastasis (Yes vs. no)	0.535	1.339 (0.533–3.362)		
Recurrence (Yes vs. no)	0.311	1.632 (0.632–4.213)		
Distant metastasis (Yes vs. no)	0.041*	3.141 (1.045–9.438)	0.47	1.663 (0.418–6.609)
Surgical approach (partial vs. total cystectomy)	0.057	2.444 (0.973–6.136)		
PGK1 expression (High vs. low)	0.007*	5.393 (1.586–18.345)	0.037*	3.768 (1.084–13.1)

*Note*: Statistical analyses were performed by Cox proportional hazards regression.

* *p* value <0.05 was considered statistically significant.

**FIGURE 2:**
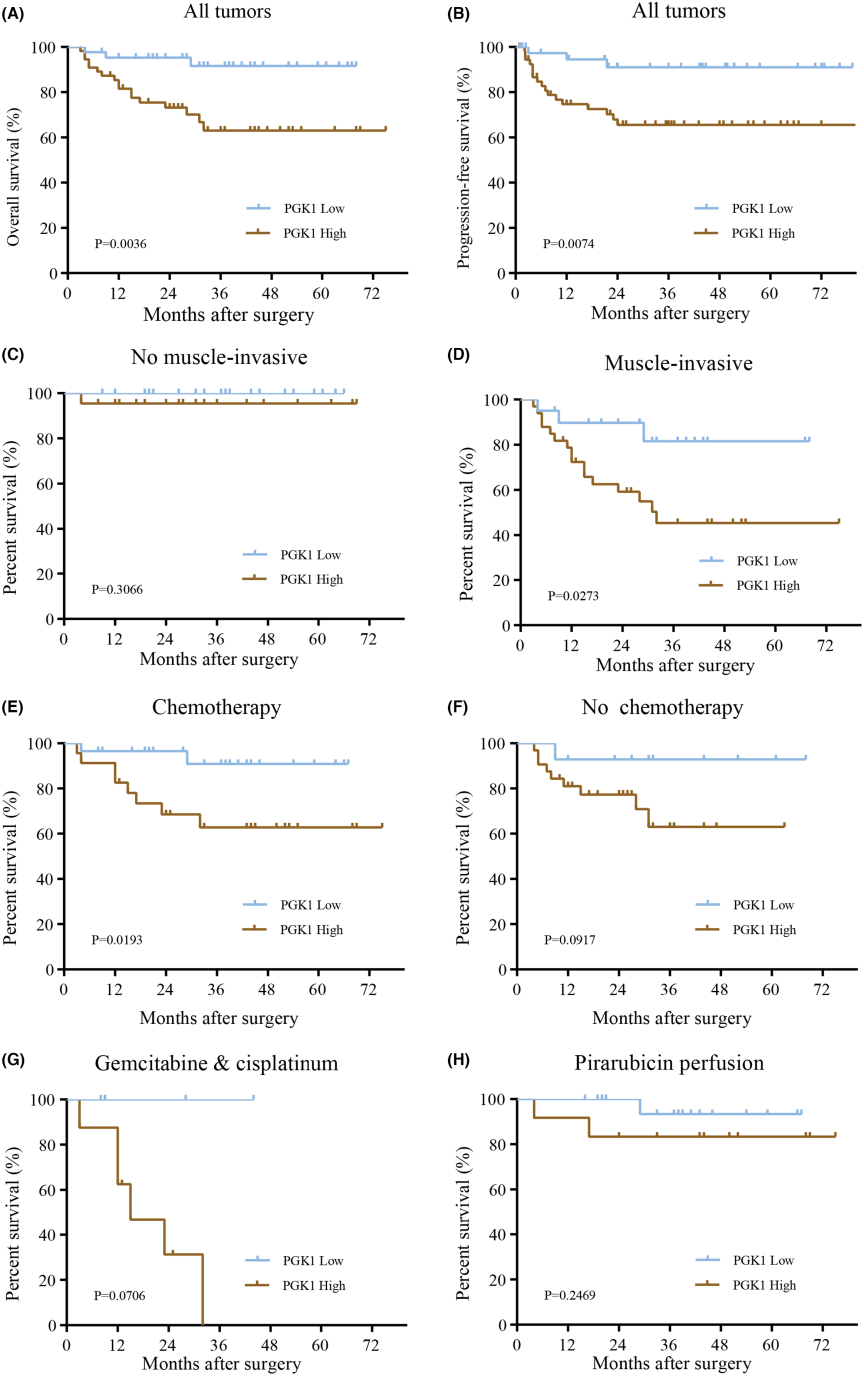
Effect of PGK1 expression on Kaplan–Meier survival curve in patients with bladder cancer (BLCA). (A, B) The high expression of PGK1 correlated with poor overall survival and progression‐free survival. (C, D) Comparison of PGK1 expression in relation to overall survival in patients with non‐muscle invasive bladder cancer (NMIBC) and muscle‐invasive bladder cancer (MIBC). (E, F) Comparison of PGK1 expression in patients treated with or without chemotherapy. (G, H) Comparison of the expression of PGK1 for patients treated with systemic chemotherapy and intravesical perfusion.

Next, the overall survival (OS) and progression‐free survival (PFS) rates were lower in BLCA patients with upregulated PGK1 compared to those with downregulated PGK1 (Figure 2A). Notably, we found that PGK1 did not affect the prognosis of NMIBC patients (Figure 2C), while upregulated PGK1 was associated with a poor prognosis in MIBC patients (Figure 2D). Additionally, we observed that PGK1 was related to the prognosis of BLCA patients who received chemotherapeutic drugs (Figure 2E) but had no impact on the prognosis of patients who did not undergo chemotherapy (Figure 2F). Furthermore, the upregulation of PGK1 was associated with reduced sensitivity to cisplatin treatment in BLCA patients (Figure 2G). However, there was no significant difference in the expression of PGK1 regarding the prognosis of patients receiving intravesical perfusion of pirarubicin (Figure 2H). Overall, these results indicate that PGK1 can serve as a prognostic factor for BLCA.

### 3.4 PGK1 can promote the proliferation of BLCA

**FIGURE 3:**
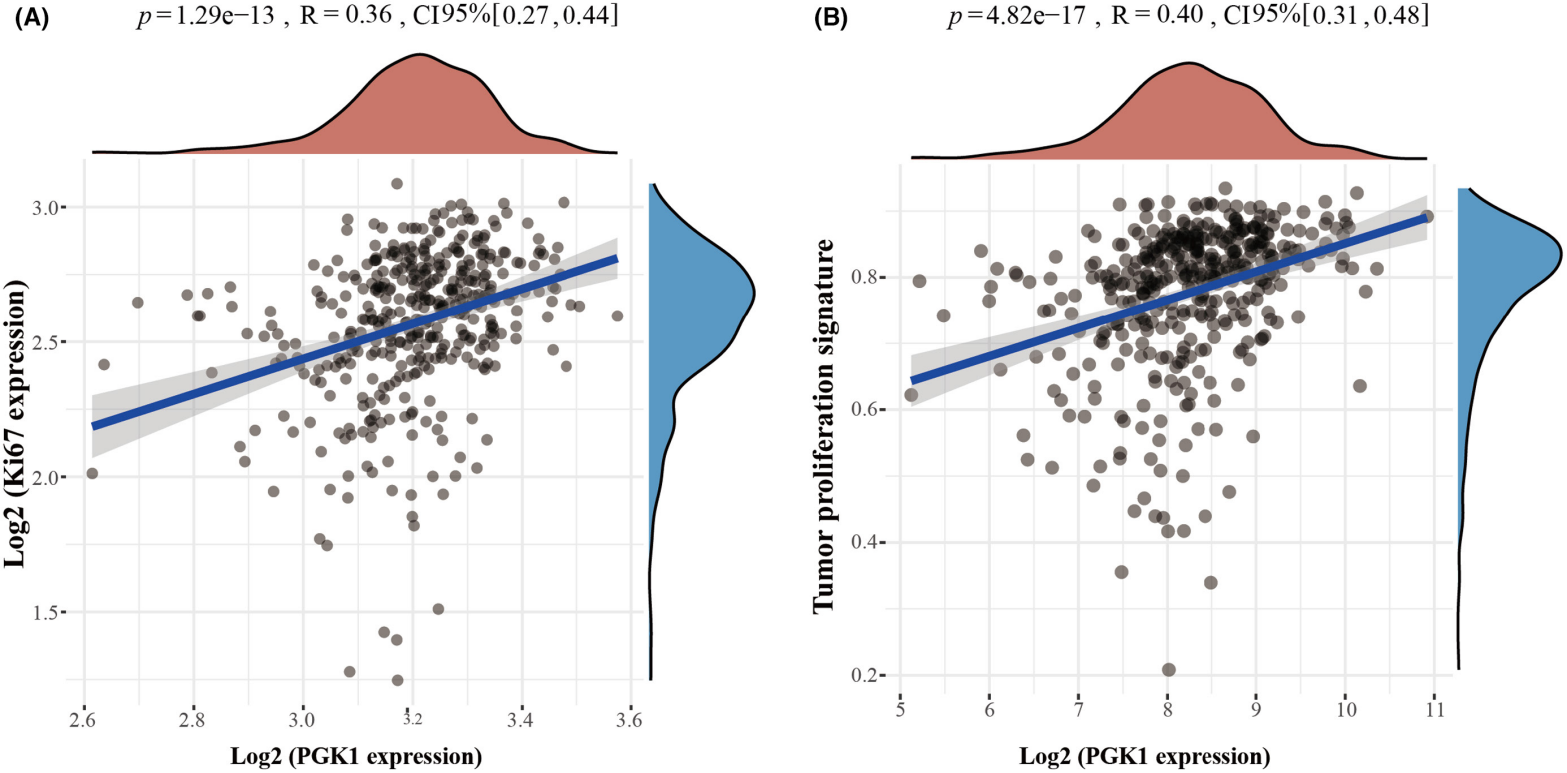
Correlation between PGK1 and proliferation index in TCGA database. (A) Correlation between the mRNA expression of PGK1 and Ki67 in TCGA database. (B) The relationship between PGK1 and tumor proliferation signature pathway in TCGA database.

To investigate the role of PGK1 in BLCA cell proliferation, we analyzed its association with Ki67, commonly used as a marker for proliferation. Previous studies conducted by our research group have examined Ki67 expression in the TMA of BLCA.^18^ Analysis of the TCGA database revealed a positive relationship between PGK1 mRNA expression and Ki67 (Figure 3A). Additionally, we found a correlation between PGK1 protein expression and Ki67 (Table 3). Moreover, using TCGA data, we calculated the enrichment fraction of each sample in the tumor proliferation pathway and demonstrated that PGK1 was associated with the tumor proliferation pathway (Figure 3B).

**TABLE 3:** Correlation between PGK1 and Ki67 expression in BLCA patients.

PGK1 expression		Ki67 expression		*p* value
		High	Low	
High	56	35	21	0.041*
Low	43	18	25	

* *p* value <0.05 was considered statistically significant.

**FIGURE 4:**
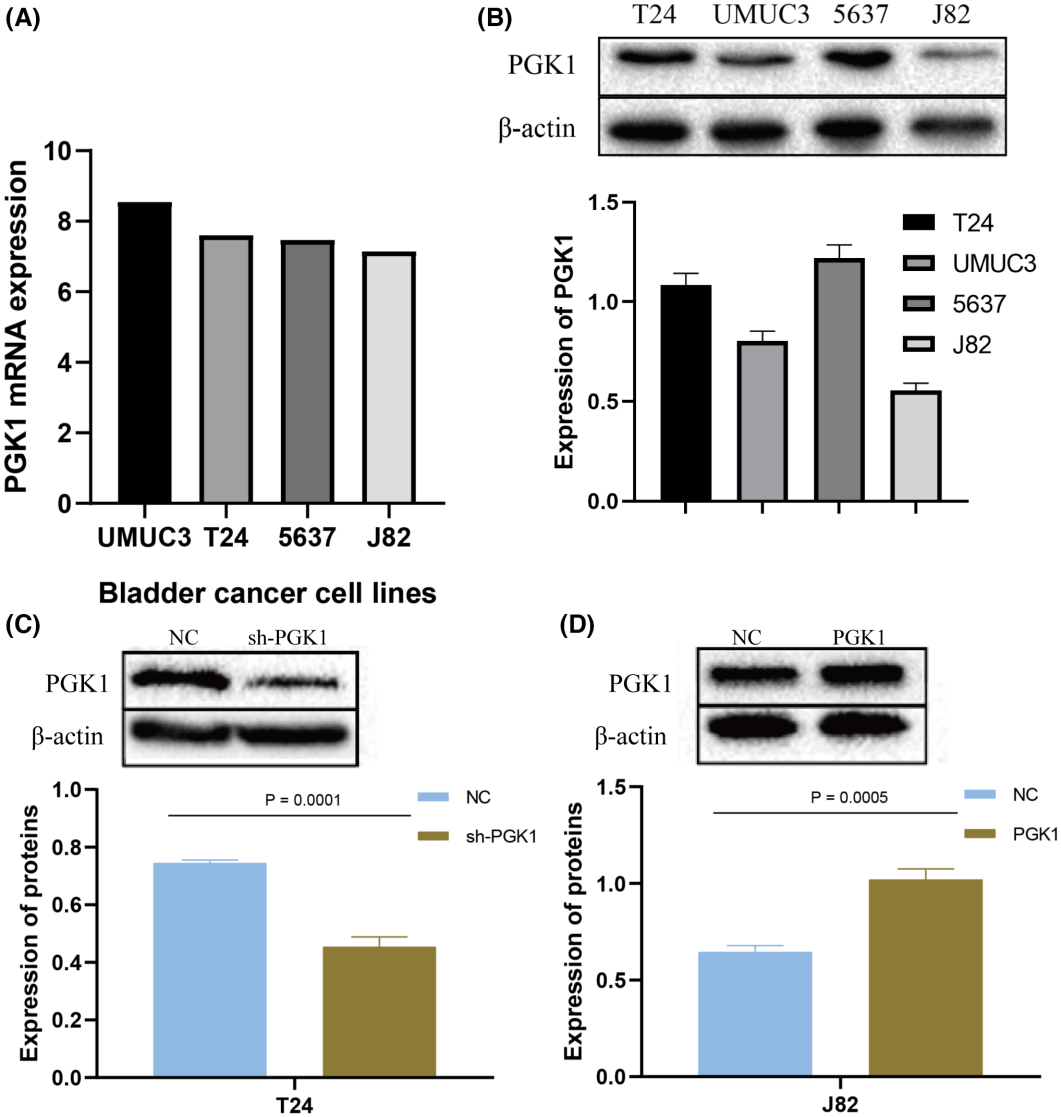
The expression of PGK1 in bladder cancer (BLCA) cell lines. (A) Exploring the mRNA expression level of PGK1 in UMUC3, T24, 5637, and J82 cell lines of BLCA in the CCLE database. (B) The protein expression level was detected by WB in UMUC3, T24, 5637, and J82 cell lines. (C) Validation of the knockdown of PGK1 in T24 cells by lentivirus. (D) Validation of the overexpression of PGK1 in J82 cells by lentivirus.

Furthermore, we explored the mRNA expression of PGK1 in T24, UMUC3, 5637, and J82 cell lines using the CCLE database (Figure 4A). The protein expression level of PGK1 was assessed through western blot analysis (Figure 4B). To comprehensively evaluate PGK1 expression, we selected T24 cells for lentivirus‐mediated knockdown and J82 cells for lentivirus‐mediated overexpression of PGK1 (Figure 4C,D).

**FIGURE 5:**
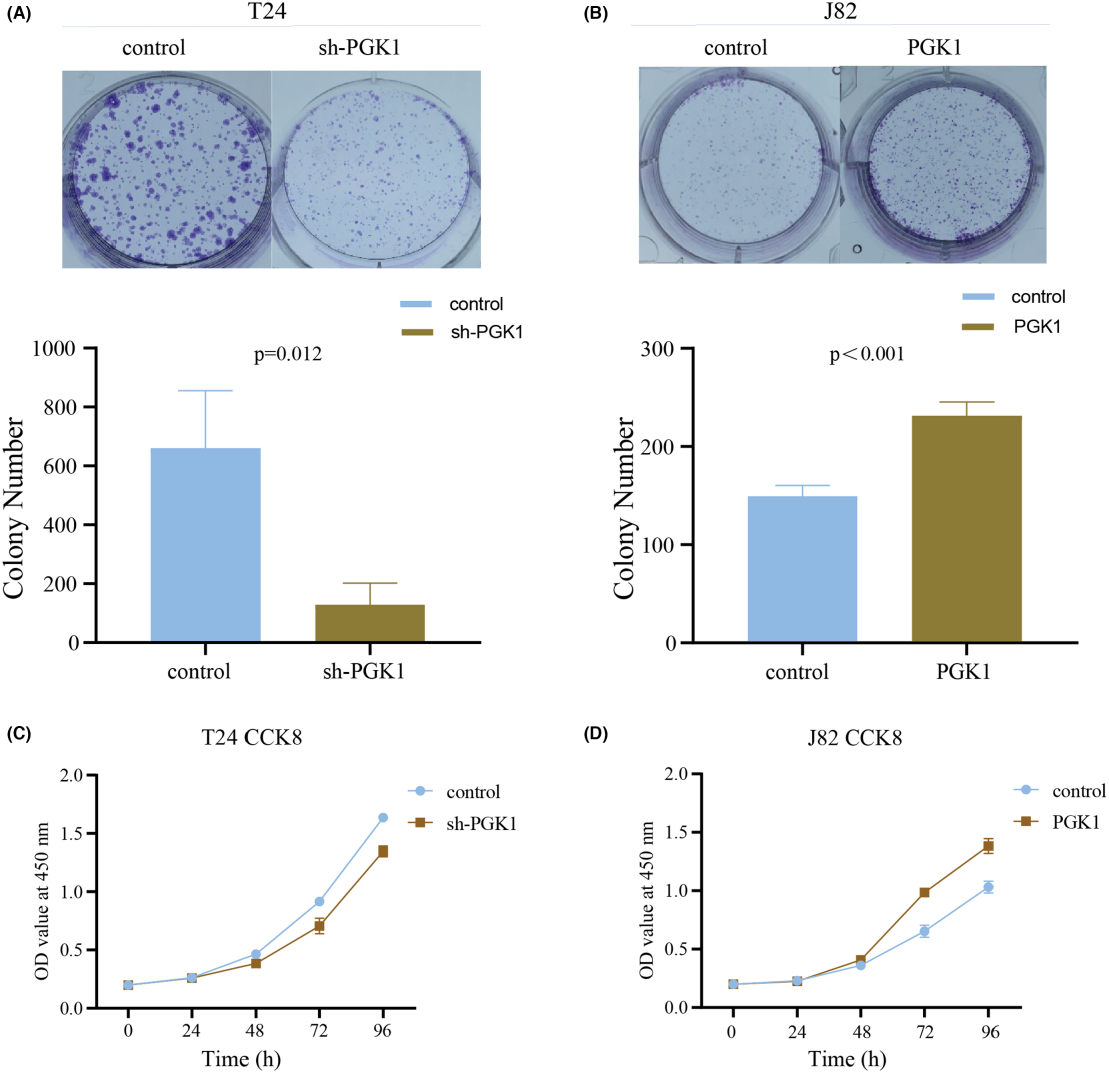
PGK1 promotes the proliferation of bladder cancer (BLCA) cells. (A) Colony formation assay demonstrated a decrease in colony formation after PGK1 knockdown. (B) Colony formation assay showed an increase in colony formation after PGK1 overexpression. (C) CCK8 proliferation assay revealed a decrease in the proliferation of T24 cells after PGK1 knockdown. (D) CCK8 proliferation assay revealed an enhancement in proliferation of J82 cells after PGK1 overexpression.

In the colony formation experiment, downregulation of PGK1 led to a reduction in colony formation (Figure 5A), while overexpression of PGK1 increased colony formation (Figure 5B). The CCK8 proliferation assay also demonstrated decreased proliferation of sh‐T24 cells (Figure 5C), while overexpression of PGK1 enhanced proliferation in J82 cells (Figure 5D). These findings indicate that PGK1 can promote the proliferation of BLCA cells.

### 3.5 PGK1 can promote metastasis of BLCA

**FIGURE 6:**
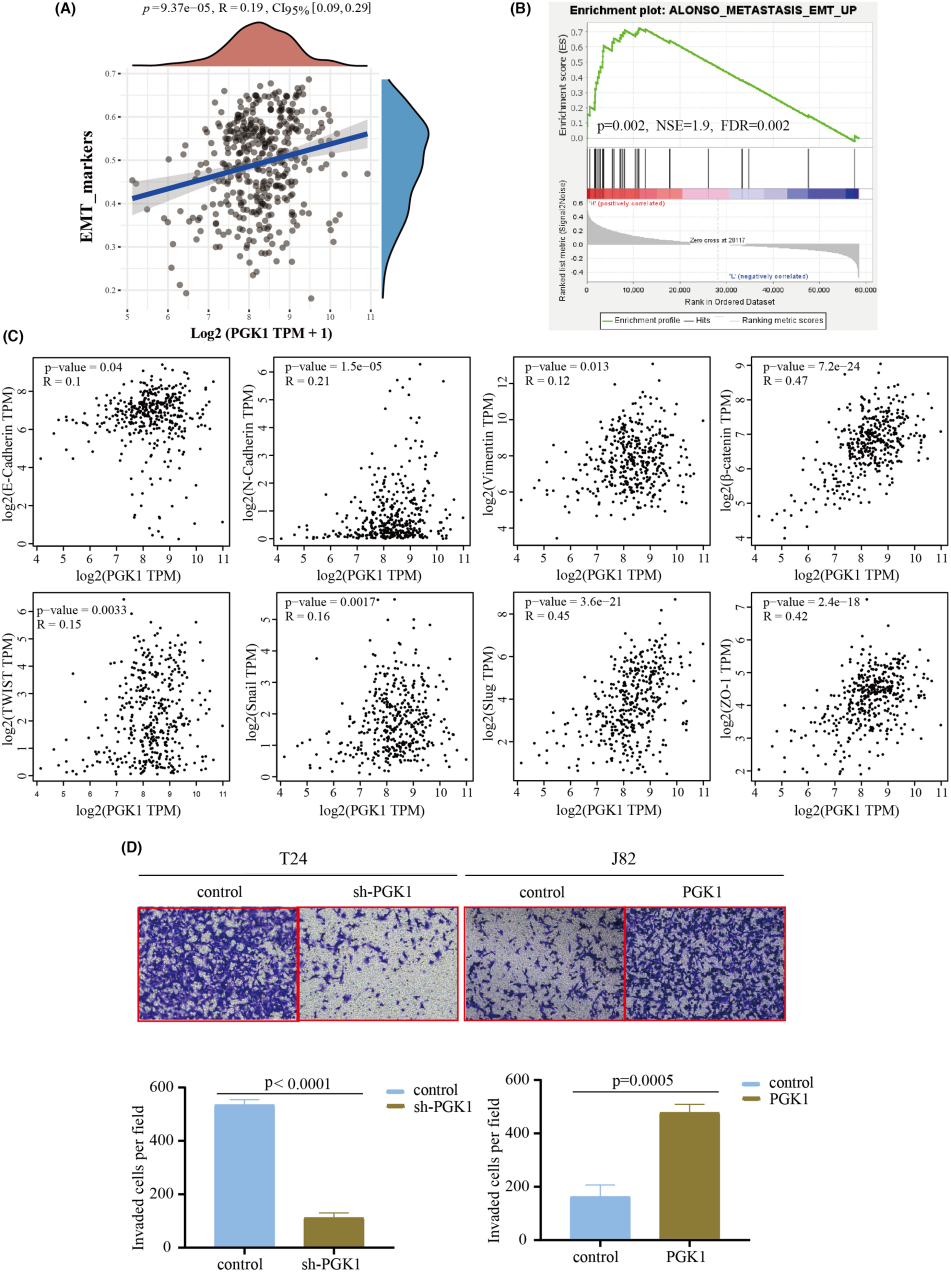
PGK1 promotes the metastasis of bladder cancer (BLCA) cells. (A) The association between PGK1 and tumor EMT markers score in TCGA database. (B) Gene Set Enrichment Analysis (GSEA) analysis indicated the involvement of PGK1 in the EMT process of BLCA. (C) The relationship between PGK1 expression and eight EMT‐related molecules was analyzed using GEPIA in the TCGA database. (D) Transwell experiments confirmed a decrease in the metastasis of T24 cells after the knockdown of PGK1 expression, while overexpression of PGK1 increased the metastasis of J82 cells.

Previous results have indicated an association between PGK1 expression and tumor invasion and lymph node metastasis. Therefore, we hypothesized that PGK1 could promote metastasis in BLCA. We observed that PGK1 was positive correlated with the EMT markers pathway in TCGA database (Figure 6A). Furthermore, Gene Set Enrichment Analysis (GSEA) revealed a significant association between PGK1 and the EMT pathway (Figure 6B). Using the GEPIA database, we directly explored the relationship between PGK1 and EMT‐related molecules, such as E‐cadherin, N‐cadherin, vimentin, β‐catenin, twist, snail, slug, and ZO‐1 (Figure 6C). Moreover, through Transwell experiments, we observed the weakened migratory capacity of sh‐T24 cells and enhanced metastatic potential of J82 cells following PGK1 overexpression (Figure 6D). These results suggest that PGK1 can promote metastasis in BLCA.

### 3.6 PGK1 was involved in cisplatin sensitivity of BLCA

Glycolysis has been implicated in the development of cisplatin chemoresistance. In sh‐T24 cells, we observed decreased glucose uptake and lactate concentration (Figure S1A,B). Conversely, overexpression of PGK1 in J82 cells increased glucose uptake and lactate concentration (Figure S1C,D). These findings suggest the involvement of PGK1 in promoting glycolysis in BLCA.

**FIGURE 7:**
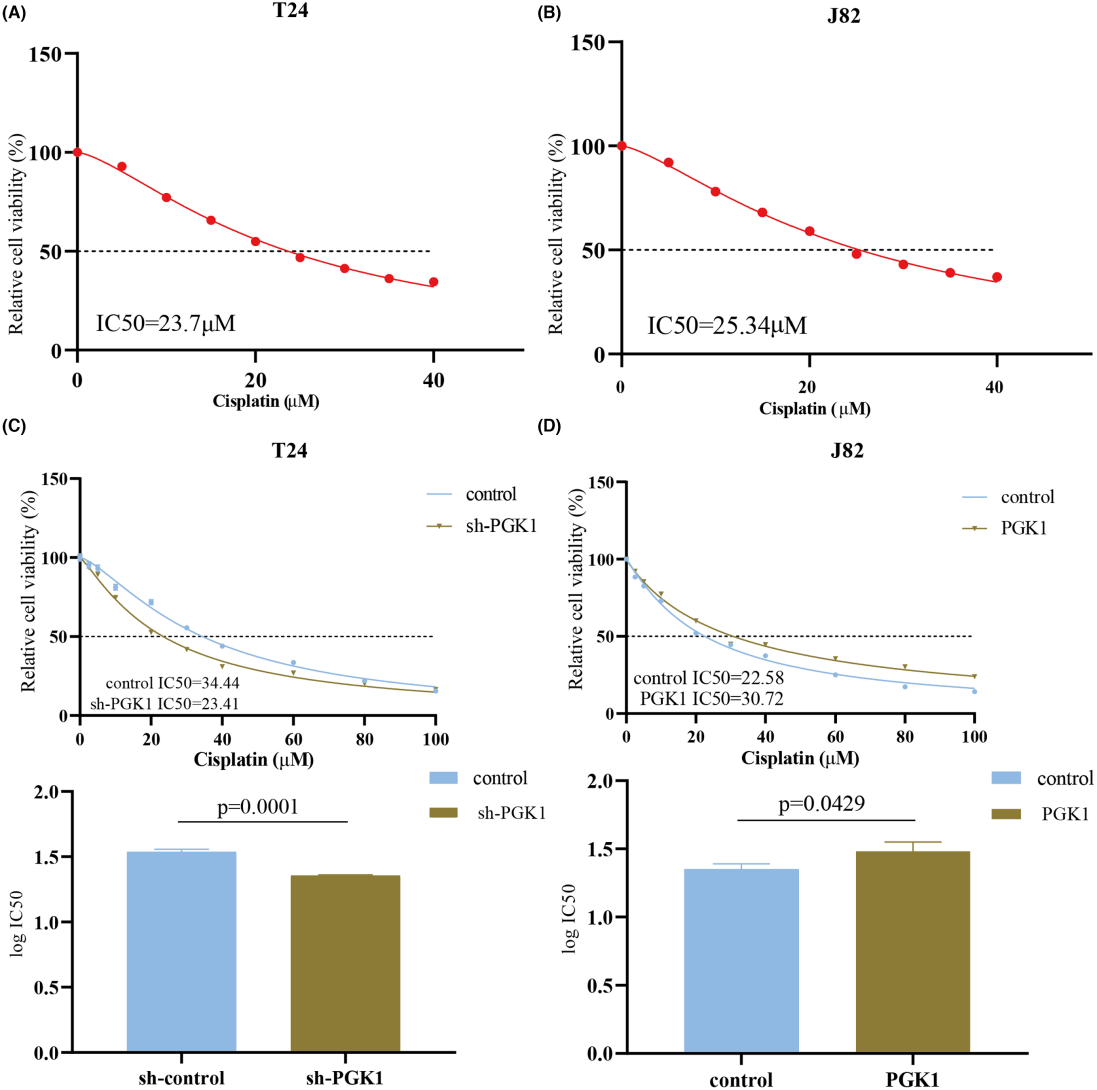
PGK1 is involved in promoting cisplatin resistance in bladder cancer (BLCA). (A, B) Cisplatin was added to T24 and J82 cells according to the concentration gradient to evaluate IC_50_. (C) IC_50_ was decreased after the knockdown of PGK1 in T24 cells. (D) IC_50_ was increased after overexpression of PGK1 in J82 cells.

Next, we treated normal T24 cells with cisplatin and determined an IC_50_ value of 23.7 μM (Figure 7A), while the IC50 for J82 cells was 25.34 μM (Figure 7B). Subsequently, we investigated changes in the IC_50_ in BLCA cells upon interference with PGK1 expression. The results showed that knockdown of PGK1 effectively reduced the IC50 (Figure 7C), while overexpression of PGK1 significantly increased the IC50 (Figure 7D).

## 4 DISCUSSION

Current evidence suggests that normal cells primarily produce ATP through oxidative phosphorylation in mitochondria under aerobic conditions, while glycolysis is inhibited by feedback from oxidative phosphorylation products. However, glycolysis offers unique advantages compared to oxidative phosphorylation. In situations where cells are deprived of oxygen due to exercise or disease, glycolysis increases to maintain a stable energy supply and generate lactic acid. Tumor cells, even in aerobic conditions, rely mainly on glycolysis to produce ATP, making glycolysis crucial for their growth and development. This metabolic phenomenon is known as aerobic glycolysis or the “Warburg effect.”^19^ PGK1, as the initial key enzyme in the glycolysis pathway, plays a role that is still poorly understood in BLCA. An increasing body of evidence suggests that PGK1 directly participates in glycolysis and oxidative phosphorylation.^15^, ^20^, ^21^ Furthermore, PGK1 has been implicated in tumor progression^15^ and the development of cisplatin chemoresistance.^13^, ^22^ O‐GlcNAc modification of PGK1 can coordinate glycolysis and tricarboxylic acid cycle, thereby promoting tumor growth.^23^ Although glycosylation has been closely linked to cisplatin resistance,^7^ the role of this protein modification of PGK1 in promoting cisplatin resistance in BLCA remains unknown. We expected further studies to confirm the relationship between PGK1 and cisplatin resistance.

This study substantiated that PGK1 promotes the proliferation and metastasis of BLCA, as well as serves as a prognostic factor for BLCA patients. However, the functions of PGK1 extend beyond these roles. PGK1 is also involved in various biological activities, including angiogenesis, autophagy, and DNA repair. For instance, PGK1 can influence autophagy by affecting the phosphorylation of Beclin1.^22^ However, its impact on angiogenesis exhibits contrasting effects on tumor development.^12^ The localization of PGK1 within cells has also been associated with different tumorigenic effects.^20^ Nevertheless, the specific functions of PGK1 in BLCA remain understudied. Therefore, exploring the potential role of PGK1 in BLCA is of great importance.

We demonstrated, through GSEA in BLCA, that PGK1 was significantly related to Pink1/Parkin, which is a classic pathway of mitophagy (Figure S2A). Inhibiting mitophagy can result in abnormal accumulation of mitochondria, which in turn reduces oxidative phosphorylation and promoting glycolysis.^24^, ^25^ Cisplatin can cause mitochondrial damage and increased ROS, leading to the upregulation of hypoxia inducible factor‐1α (HIF‐1α).^26^ HIF‐1α can regulate mitophagy by increasing the expression of mitochondrial outer membrane‐related receptors.^26^, ^27^, ^28^ Mitophagy can also contribute to cisplatin chemoresistance in tumors.^29^ Therefore, we used the Molecular Signatures Database (MSigDB) to analyze mitophagy‐related genes, and further screened the molecules most related to PGK1 through PPI (protein–protein interactions). Finally, we determined that there might be an interaction between voltage dependent anion channel 1 (VDAC1) and PGK1 (Figure S2B,C). VDAC1 can regulate the permeability and integrity of the mitochondrial outer membrane,^30^ and it can combine with hexokinase, the rate‐limiting enzyme of glycolysis, to promote a highly glycolytic phenotype and chemoresistance in tumors. Therefore, further exploration of the interaction between PGK1 and VDAC1 is also warranted. Parkin, an E3 ubiquitin protein ligase, can be phosphorylated by PINK1, translocated to the outer membrane of mitochondria, ubiquitinate VDAC1, and finally leading to mitophagy.^31^ Dephosphorylation of VDAC1 can lead to subsequent mitochondrial permeability leakage, ultimately resulting in the release of cytochrome c and apoptosis.^32^ Therefore, we hypothesize that PGK1 can directly phosphorylate VDAC1 and counteract the ubiquitination of VDAC1 mediated by Parkin.

By searching Gene‐Cloud Biotechnology Information (GCBI), we have successfully predicted that signal transducer and activator of transcription‐5A (STAT5A) may function as a tumor‐promoting transcription factor of PGK1. Furthermore, our findings indicate the involvement of STAT5A in BLCA's glycolysis and mitophagy through GSEA. It has been reported that STAT5A can translocate into mitochondria and inhibit oxidative phosphorylation.^33^ We predict that the direct phosphorylation of PGK1 in mitochondria can both enhance the transcription of PGK1 and prevent the direct oxidation. According to the characteristics of signal transducer and activator of transcription‐3 (STAT3),^27^, ^34^ we speculate that STAT5A can work together with HIF‐1α and are also involved in mitophagy. In conclusion, due to the existence of hypoxic microenvironment in BLCA, cisplatin chemotherapy exacerbates the hypoxic state, resulting in co‐activation of STAT5A and HIF‐1α on PGK1. Subsequently, PGK1 interacts with and phosphorylates VDAC1, thereby enhancing the stability of the mitochondrial structure, and combating Pink1/Parkin/VDAC1‐mediated mitophagy, which leads to cisplatin resistance in BLCA.

We hope to improve relevant experiments in the future and further prove the correctness of our speculation on the new cisplatin resistance mechanism (Figure S2D). In addition, the animal experiments were not conducted to verify the effect of PGK1 expression on cisplatin treatment for BLCA. Although our study suggested that PGK1 could serve as a prognostic factor in BLCA, the area under the curve (AUC) for PGK1 detection was only 0.687. We acknowledge that this finding may be attributed to the limitation of sample size, and therefore, larger datasets are required to validate our conclusions in future studies. Therefore, further experimental investigations are warranted to elucidate the precise role of PGK1 in BLCA and provide valuable insights into the clinical implications of cisplatin therapy.

## AUTHOR CONTRIBUTIONS

**Mingde Gao:** Formal analysis (equal); funding acquisition (equal); methodology (equal). **Haixia Zhu:** Formal analysis (equal); investigation (equal); writing – original draft (equal). **Haifei Xu:** Formal analysis (equal); supervision (equal); validation (equal). **Xiaoxia Jin:** Methodology (equal); supervision (equal); writing – review and editing (equal). **Guihua Zheng:** Methodology (equal); resources (equal); software (equal). **Jinfeng Zhu:** Data curation (equal); visualization (equal). **Chunyan Gu:** Funding acquisition (equal); project administration (equal); supervision (equal); writing – review and editing (equal). **Xiaolin Wang:** Conceptualization (equal); project administration (equal); visualization (equal).

## FUNDING INFORMATION

This research was funded by Nantong Science and Technology Project Fund (JC22022025) and Nantong Municipal Health Commission Project Fund (MS2022069 and MSZ2023036). The funding provided by these organizations has contributed to the completion of this study, including the coverage of publishing costs for this article.

## CONFLICT OF INTEREST STATEMENT

The authors declare that they have no conflicts of interest in this work.

## ETHICS STATEMENT

This study has been approved by Nantong Tumor Hospital Medical Ethics Committee (No. 023‐A 08).

## CONSENT

The oral consent was obtained from the patients for the use of their tissues in research.

## Supporting information


Figure S1:
Figure S2:

## Data Availability

All data comes from public databases, and there are links in the corresponding places of the article. The TMA data were obtained from our hospital, while patient survival information was acquired from the local public security bureau. Besides, the data generated involved in the research are available from the corresponding author upon reasonable request.
