# Supplementary material for: PGK1 can affect the prognosis and development of bladder cancer
Source: Cancer Med. 2024 Sep 24;13(18):e70242. doi: 10.1002/cam4.70242 (PMC11420942; doi:10.1002/cam4.70242)
Supplement: Supplementary file 1 — Figure S1: Figure S2: [file CAM4-13-e70242-s001.docx]

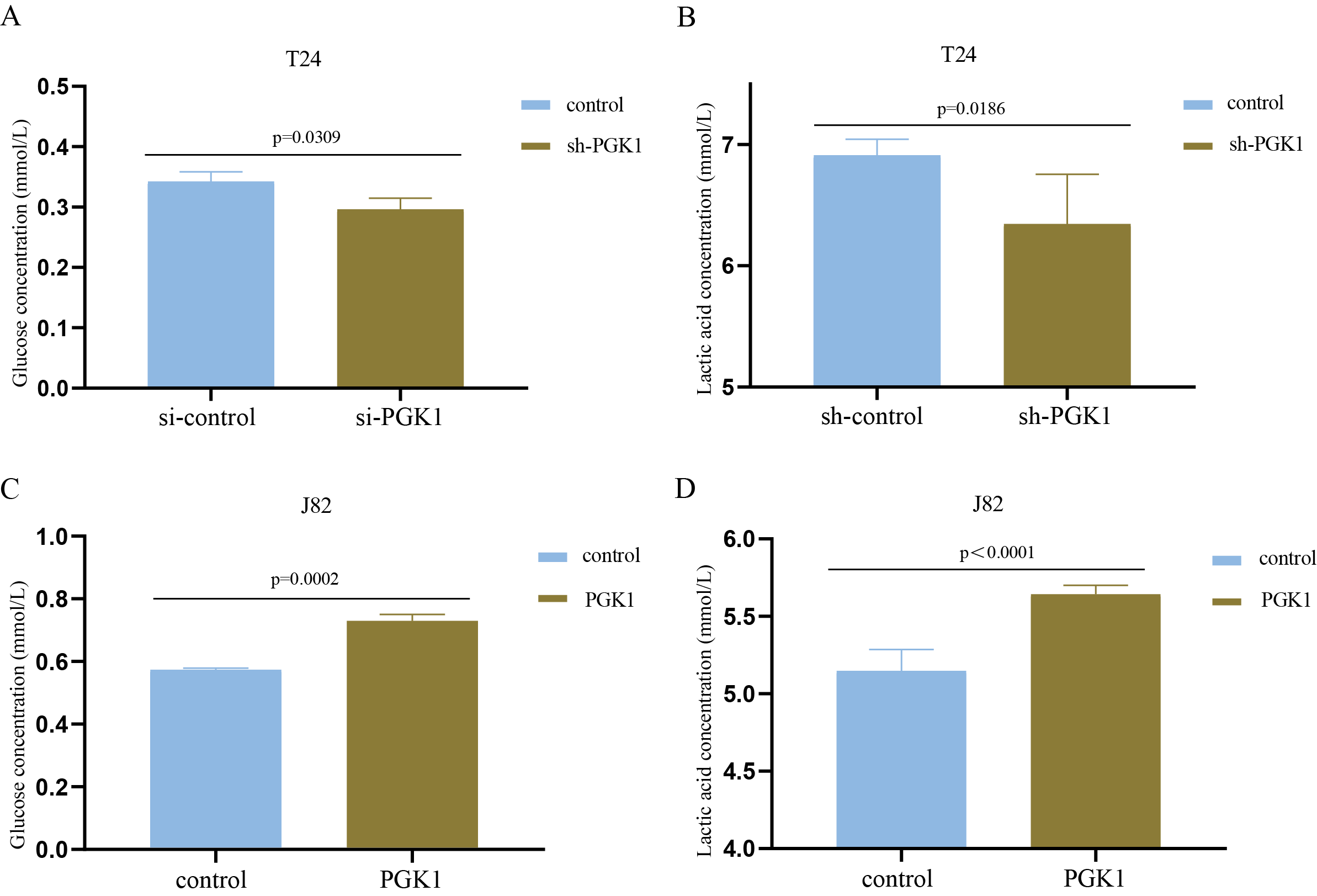


**Supplementary Figure 1: PGK1 was involved in regulating the glycolysis of bladder cancer.** **(A)** In T24 cells, the knockdown of PGK1 reduced the glucose uptake ability. **(B)** In T24 cells, the knockdown of PGK1 can reduce the lactic acid concentration. **(C)** In J82 cells, overexpression of PGK1 can enhance glucose uptake. **(D)** In J82 cells, overexpression of PGK1 can increase lactate concentration.


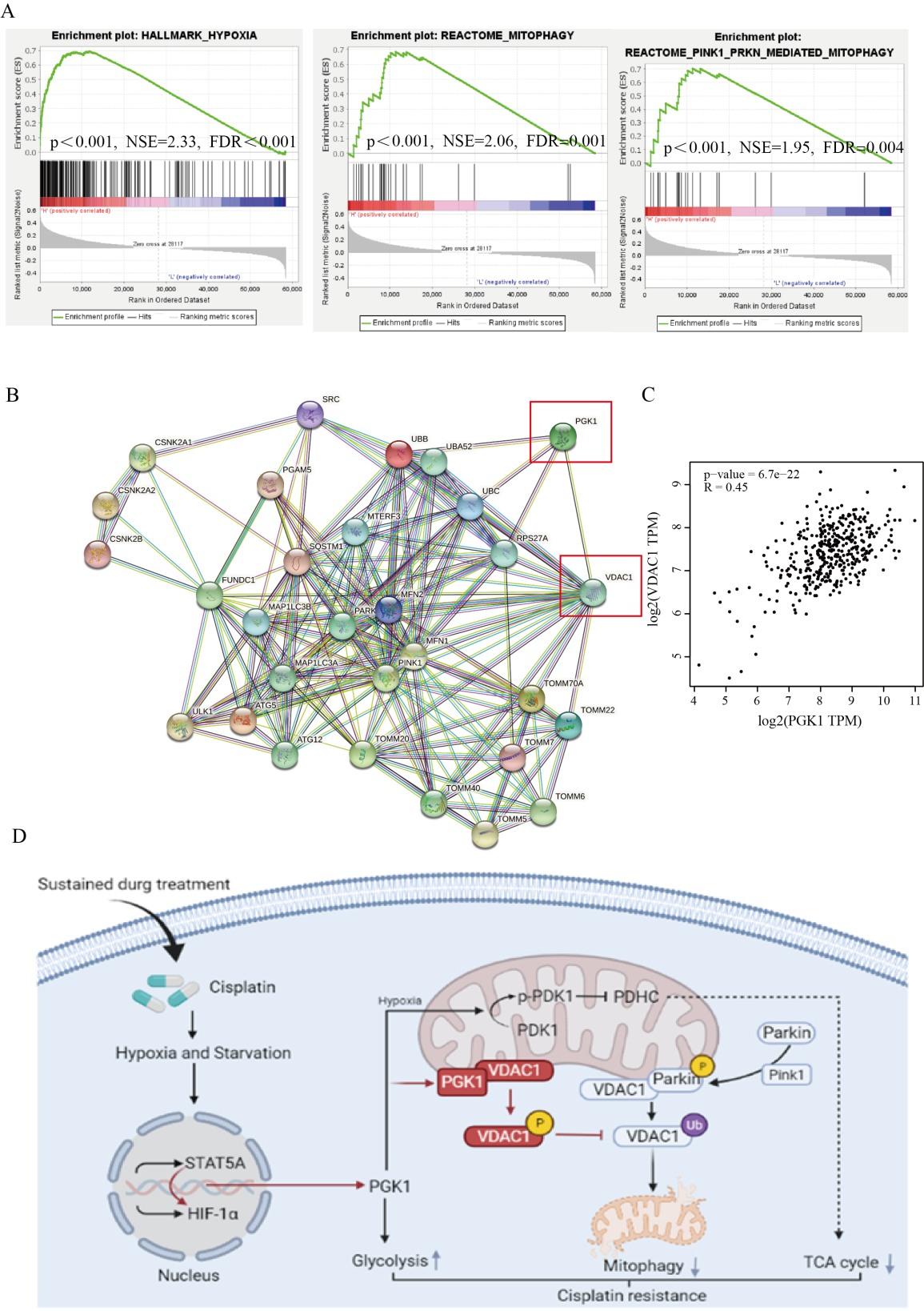


**Supplementary figure 2: bioinformatics analysis of PGK1 involved in the potential mechanism of cisplatin resistance in bladder cancer.** **(A)** The potential mechanism of PGK1 involved in cisplatin resistance was analyzed in bladder cancer by GSEA. **(B)** Protein interaction network was used to analyze the interaction between PGK1 and mitophagy-related protein VDAC1. **(C)** TCGA database verified the correlation between PGK1 and VDAC1. **(D)** PGK1 was involved in the potential mechanism of cisplatin resistance in bladder cancer.
